# Prospects and challenges of dynamic DNA nanostructures in biomedical applications

**DOI:** 10.1038/s41413-022-00212-1

**Published:** 2022-05-23

**Authors:** Taoran Tian, Yanjing Li, Yunfeng Lin

**Affiliations:** 1 State Key Laboratory of Oral Diseases, National Clinical Research Center for Oral Diseases, West China Hospital of Stomatology, Sichuan University, Chengdu, 610041 P. R. China; 2 Department of Prosthodontics, Tianjin Medical University School and Hospital of Stomatology, Tianjin, 300070 P. R. China

**Keywords:** Bone

## Abstract

The physicochemical nature of DNA allows the assembly of highly predictable structures via several fabrication strategies, which have been applied to make breakthroughs in various fields. Moreover, DNA nanostructures are regarded as materials with excellent editability and biocompatibility for biomedical applications. The ongoing maintenance and release of new DNA structure design tools ease the work and make large and arbitrary DNA structures feasible for different applications. However, the nature of DNA nanostructures endows them with several stimulus-responsive mechanisms capable of responding to biomolecules, such as nucleic acids and proteins, as well as biophysical environmental parameters, such as temperature and pH. Via these mechanisms, stimulus-responsive dynamic DNA nanostructures have been applied in several biomedical settings, including basic research, active drug delivery, biosensor development, and tissue engineering. These applications have shown the versatility of dynamic DNA nanostructures, with unignorable merits that exceed those of their traditional counterparts, such as polymers and metal particles. However, there are stability, yield, exogenous DNA, and ethical considerations regarding their clinical translation. In this review, we first introduce the recent efforts and discoveries in DNA nanotechnology, highlighting the uses of dynamic DNA nanostructures in biomedical applications. Then, several dynamic DNA nanostructures are presented, and their typical biomedical applications, including their use as DNA aptamers, ion concentration/pH-sensitive DNA molecules, DNA nanostructures capable of strand displacement reactions, and protein-based dynamic DNA nanostructures, are discussed. Finally, the challenges regarding the biomedical applications of dynamic DNA nanostructures are discussed.

## Introduction

Thirty years ago, when the understanding of DNA was mainly focused on its role in transmitting information as a genetic material, Professor Nedrian Seeman pioneered the use of DNA sequences as building blocks to construct structures, opening up a new field of research in DNA nanotechnology.^1,2^ DNA possesses excellent biocompatibility owing to its abundant existence in the human body. From their initial recognition, DNA nanostructures have been considered highly editable because of the structural chemistry of deoxyribonucleic acids and the sequence diversity provided by the four bases.^3,4^ Initially, due to the lack of computer-aided design tools, research on DNA structures was mainly limited to small structures, for example, the exploration of the properties of DNA structures,^5,6^ framework nucleic acid structures,^7,8^ random DNA coils, and two-/three-dimensional structures composed of DNA tiles.^9,10^ In the past decade, the development of computer-aided technology has allowed enormous advances in related research.^11^

**Fig. 1:**
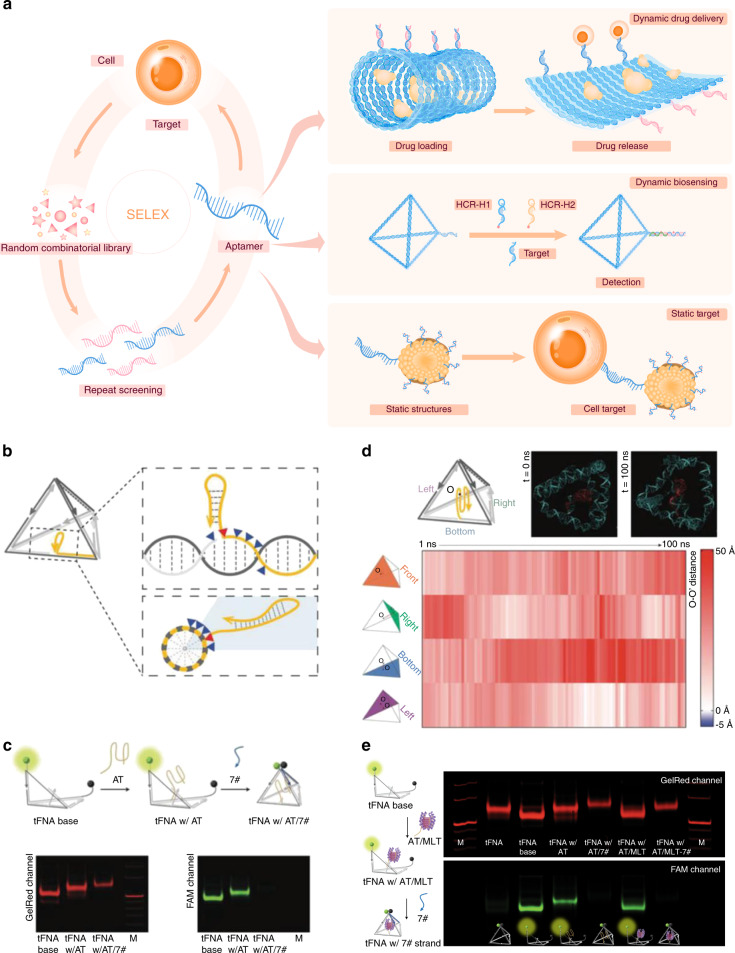
Dynamic DNA structures based on DNA aptamers. **a** Schematic illustration of the screening process for aptamer and aptamer-based nucleic acid nanostructures. **b** Continuous sites were screened for cargo loading via PAGE (screened sites are denoted as blue triangles). The best loading site (shown in the panel, red triangle) was selected by lagging tag modification (yellow loop at the 3′ end). **c** The cargo loading strand (attachment strand, AT strand) was loaded into the exoskeleton with stepped annealing: the green sphere represents the 3′ modification of FAM, the black sphere represents the 5′ modification of BHQ-1. The fluorescence changes and band shifts in the PAGE gel indicated successful fabrication of the AT strand-loaded tFNA exoskeleton. **d** All-atom MD simulation of the AT strand-loaded tFNA exoskeleton revealed that the AT strand was well encapsulated inside the tFNA exoskeleton. The distances between the center of mass of the G-quadruplex strand (O) and the four surfaces of the tFNA (O-O′) were calculated to be positive in the equilibrium state. **e** The MLT-loaded tFNA exoskeleton, the nanobee, was fabricated via three-step annealing and verified by PAGE gel electrophoresis.^113^ Copyright 2022, John Wiley and Sons

**Fig. 2:**
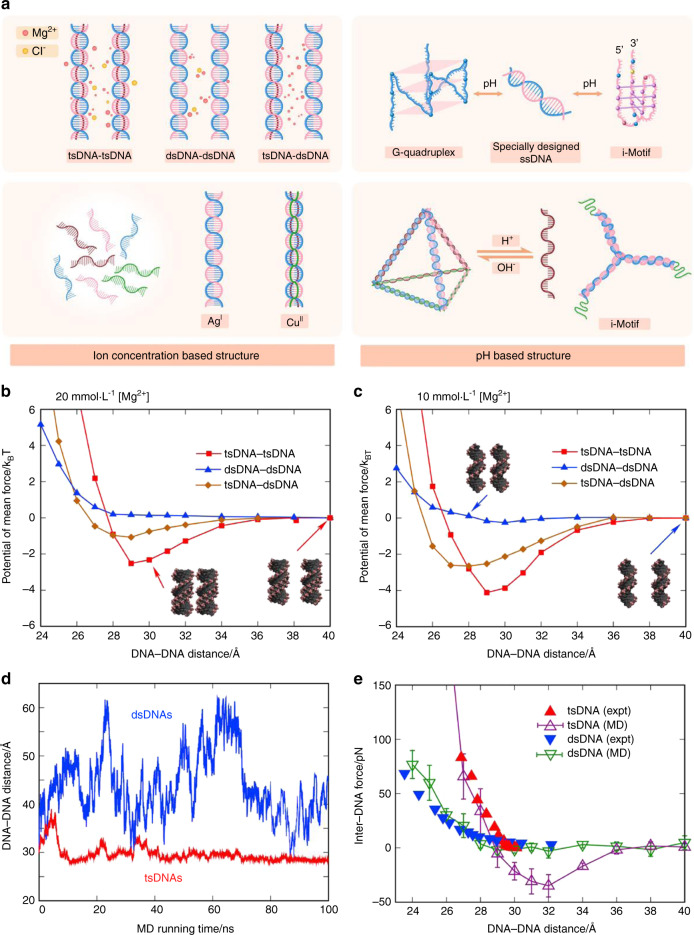
Dynamic DNA structures based on the ion concentration/pH. **a** Schematic illustration of dynamic DNA structures based on the ion concentration or pH. The mean force potentials between DNAs are given for three cases, namely, tsDNA-tsDNA, dsDNA-dsDNA, and tsDNA-dsDNA, in ∼20 (**b**) and ∼100 mmol·L^−1^ Mg^2+^ solutions (**c**). **d** Shown here is the distance between the mass center of the DNAs versus the MD simulation time from MD simulations for two tsDNAs (red) and two dsDNAs (blue) without the connection spring in a 20 mmol·L^−1^ Mg^2+^ solution. **e** The inter-DNA forces were calculated from our MD simulations and the experimental measurements on osmotic pressure.^151^ Copyright 2017, Biophysical Journal

**Fig. 3:**
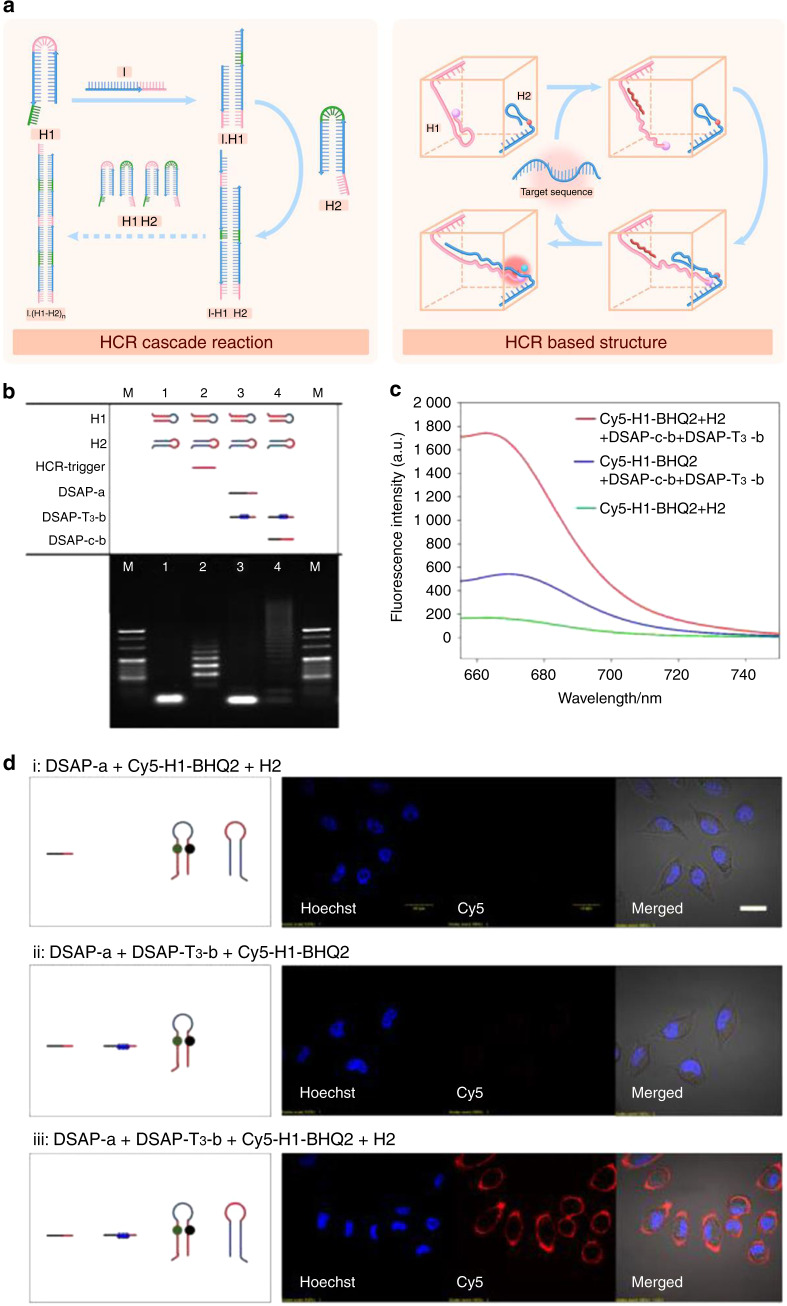
Dynamic DNA structures based on strand displacement reactions. **a** Schematic illustration of dynamic DNA structures based on the strand displacement reaction. **b** The gel electrophoresis and **c** fluorescence characterization results of the HCR products induced by a DSAP using DSAP-c-b to simulate target recognition. Lane M: DNA marker. **d** Fluorescence confocal microscopy images of target SMMC-7721 cells after incubation with different DNA probes. (i) DSAP-a + Cy5-H1-BHQ2 + H2, (ii) DSAP-a + DSAP-T_3_-b + Cy5-H1-BHQ2, and (iii) DSAP-a + DSAP-T_3_-b + Cy5-H1-BHQ2 + H2. Cells were stained with Hoechst 33342 before imaging. (Scale bar: 20 μm).^89^ Copyright 2020, American Chemical Society

**Fig. 4:**
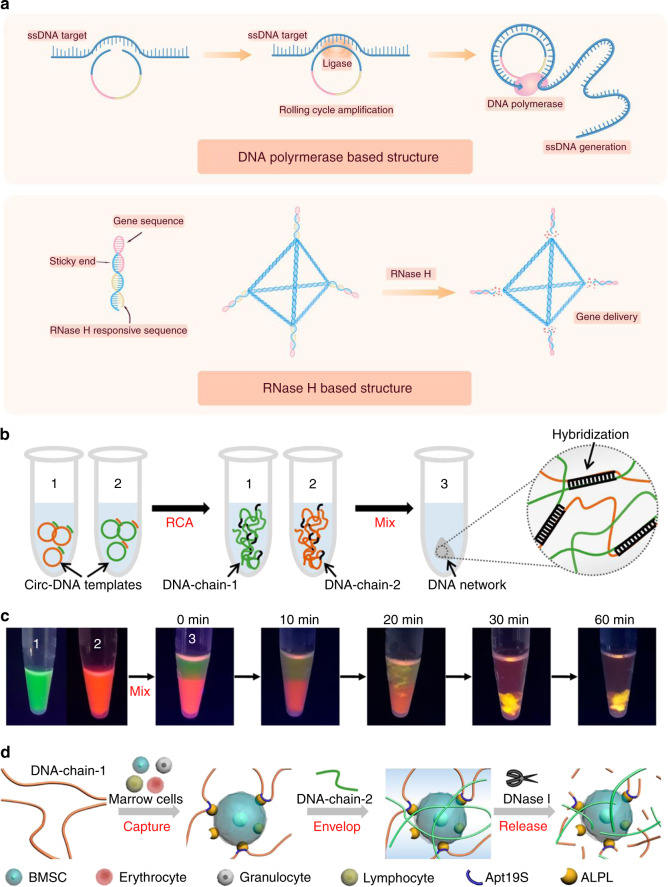
Dynamic DNA structures based on proteins or other substances. **a** Schematic illustration of dynamic DNA structures based on proteins. **b** Synthetic process of ultralong DNA chains via RCA to obtain a 3D DNA network. **c** Mix of DNA chains to visualize the molecular diffusion and phase inversion during the formation of a DNA network. DNA-chain-1 and DNA-chain-2 were stained with SYBR Green II and Gel Red, respectively. **d** The process of capture, envelopment, and release: (1) capture, DNA-chain-1 was incubated with BMSCs for cell capture by anchoring to the aptamer Apt19S; (2) envelopment, DNA-chain-2 was added to the cell-containing DNA-chain-1 solution to trigger the formation of the DNA network; and (3) release, the DNA network can be digested by the nuclease DNase I to release BMSCs.^152^ Copyright 2020, American Chemical Society

Douglas reported a game-changing open-source software package, caDNAno, which focused on the double helix scale and chiral rotation of the DNA molecule itself and aided in designing DNA sequences for the construction of 3D honeycomb-pleated shapes.^12^ Tiamat is an efficient three-dimensional editing tool that focuses on the topological structure and complementary pairing relationship of DNA.^13^ Zadeh et al. reported a nucleic acid package (Nupack) for thermodynamic analysis of dilute solutions of interacting nucleic acid strands and tube simulation.^14^ Cando and oxDNA have been used for coarse molecular dynamics analysis of designed DNA structures.^15,16^ MagicDNA, recently proposed by Castro’s group, integrates multiple functions and possesses a visual interface. These tools allowed the development of a large number of new DNA structures, such as DNA origami, by which long ssDNA is folded with short ssDNA molecules used as staples;^17^ DNA frameworks, generated by folding a series of short ssDNA molecules;^18^ dynamically triggered DNA networks composing tetrahedral nanostructures;^19^ and large-scale two- or three-dimensional DNA arrays self-assembled by DNA tiles.^20^ These structures constitute the necessary conditions for the application of DNA nanostructures. Figures 1–4.

Recently, various DNA nanostructures have been applied in a wide array of fields. Due to their great editability, DNA nanostructures can be designed and synthesized with excellent predictability at the nanoscale.^21^ Because of this property, DNA nanostructures are used as tools in various fields, such as biocomputing, bioimaging, biomimetic structure construction, and drug delivery.^22^ There are two main application forms of DNA nanostructures: static and dynamic. A static structure refers to a structure constructed directly from DNA molecules, which accomplishes multiple functions through the physical and chemical properties of the DNA structure itself, such as its physical structure, size, charge, and nucleic acid composition. Among of the various static structures, DNA tetrahedral nanostructures are widely applied due to their simple synthesis, relatively high yield, and high economic performance. Professor Yunfeng Lin and coworkers from Sichuan University applied a DNA tetrahedral nanostructure (tFNA) to a variety of disease models, and in vivo and in vitro experiments showed that tFNA can intervene in the process of disease to varying degrees.^23^ The material possesses outstanding anti-inflammatory and antioxidant abilities, and it can ameliorate a variety of conditions whose main pathological manifestation is inflammation-related destruction, such as skin injury, acute kidney injury, and osteoarthritis.^24–29^ On the other hand, DNA nanostructures, as building blocks, can be used to construct a variety of static structural forms and are used in various research fields.^30–32^ For example, Chenxiang Lin’s group from Yale University built a DNA cylinder with a pore size of ~30 nm and modified it with nuclear pore proteins to mimic nuclear pore complexes in vitro, thus allowing the properties and function of this complex to be studied.^33,34^ Static DNA nanostructures have great application potential and value. However, one of the core advantages of DNA nanostructures lies in their valuable application functions endowed by ingenious and flexible dynamic DNA structure design. Based on DNA aptamers, G-quadruplexes (G4s), hairpin structures, binding enzymes, or other proteins, DNA structures can respond to the external environment. For example, Damien et al. reported a “DNA walker” based on a static DNA assay and hairpin structure and having the front-end input ability for DNA computing.^35^ In the biomedical field, the response of DNA to the external environment is mainly exploited in drug delivery systems based on DNA nanostructures. Ding et al. utilized a DNA origami sheet to construct a thrombin or immune antigen delivery system.^36,37^ The dynamic and static DNA structures are complementary to each other, yet in addition to relying on the static DNA structure, the dynamic structure can be used as a unique modification in other materials. For instance, dynamic DNA structures can be covalently linked to other polymers to form environmentally sensitive hydrogels^38^ or linked to nanogold particles to create specific labels.^39^

At present, research on DNA nanostructures is mainly focused in two directions. One is the synthesis of arbitrary DNA structures, for example, the computer-aided synthesis of large-scale arbitrary two-dimensional and three-dimensional DNA structures proposed by Qian, Dietz, and Yin et al.^20,40–42^ The other is the application of various dynamic structures. For example, i-motifs with different conformations under different pH conditions are used to design pH-responsive vectors,^43–47^ unique DNA sequences are utilized to target specific proteins or DNA sequences and accomplish dynamic structural functions,^36,48,49^ DNA hairpin groups are employed to realize the polymerization chain reaction of specific sequences,^31,50,51^ specially designed three-dimensional nucleic acid structures are used to realize temperature-sensitive drug delivery,^52,53^ and DNA structures are used in combination with DNA polymerase as the template for rolling circle replication/rolling circle amplification (RCR/RCA) to construct dynamic drug delivery systems.^54–56^ All the above findings reveal the broad application prospects of dynamic DNA structures in the biomedical field. With this in mind, this review aims to review the structural basis of dynamic DNA nanostructures, summarize the application status of DNA nanostructures in the biomedical field, and discuss the future application prospects of DNA nanostructures.

## Chemical and structural basis of dynamic DNA nanostructures

### Dynamic DNA structures based on DNA aptamers

Complementary pairing allows nucleic acids to form double strands, as well as a great diversity of complex structures, which can interact with biological molecules such as proteins. Various oligonucleotides can regulate the biological behaviors of proteins by regulating their activity.^57^ In addition to the biological interactions between oligonucleotides and biological molecules, aptamers, another type of single-stranded nucleic acids, can bind to proteins via nonbiological mechanisms.^58–60^ Structurally, aptamers are based on single-stranded DNA (ssDNA) or RNA (ssRNA), which is mainly selected by a program named sequential evolution of ligands by exponential enrichment (SELEX). With this technique, the single-stranded nucleic acid sequence with the best specificity and highest binding strength for targets can be selected from an extensive oligonucleotide library (including 10^13^−10^15^ different sequences of 20–100 nt).^61^ Depending on the aptamer screening method, the only function of aptamers is to achieve specific binding to targets, and the binding strength of aptamers is between 0.000 2 and 1 100 K_d_ (nmol·L^−1^) for diverse targets.

Although aptamers possess different sequences and structures, they can form multiple secondary structures, such as double strands, DNA loops, and G4 structures, by self-assembly to achieve targeted binding with different biological molecules. Since SELEX technology was proposed in 1990, aptamers have been shown to have high affinity for various protein families, including but not limited to cytokines, proteases, kinases, cell surface receptors, and cell adhesion factors. Aptamers exert certain biological effects through this combination of properties. It is widely believed that the binding of an aptamer to a protein indeed inhibits the interaction of the protein with other biomolecules, thereby affecting its function. The aptamer pegaptanib is used to target vascular endothelial growth factor (VEGF) and abolish its function, and pegaptanib has been approved for the treatment of age-related macular degeneration.^62^ Another aptamer, AS1411, is utilized to target nucleolin, which is highly expressed on the surface of cancer cells,^63^ thereby functionally inhibiting tumor cells and treating acute myeloid leukemia (phase II clinical trial).^64^ In addition to inhibiting protein function through direct binding, researchers have also developed various drug delivery systems by targeting cells with high-affinity aptamers. Simple applications include the direct use of aptamers to achieve targeting, or to permit or enhance the binding of the drug delivery system to the protein.^65^ In addition, aptamers can accomplish dynamic functions by delicate design. Han et al. developed nucleolin-sensitive drug delivery system exploiting the affinity of AS1411 for nucleolin.^66^ The system is stable in the circulation by binding to its complementary chain while breaking the chain when the system is exposed to nucleolin; then, AS1411 binds to nucleolin, which opens the drug delivery system and releases the drug. This dynamic structure and function depend on the different affinities between the aptamer and different structures. Through such chain replacement reactions, downstream dynamic reactions, such as hybridization chain reaction (HCR), can be further activated to obtain much more diverse dynamic structural designs.

### Dynamic DNA structures based on the ion concentration/pH

Since the double strands of nucleic acids in DNA are connected by hydrogen bonds, the structure of nucleic acids can be affected by the ion concentration and environmental pH. It has been confirmed that various ions can bind to single- or double-stranded nucleic acids and act as a medium to induce conformational changes or generate bridges to form complex structures.^67–69^ Mg^2+^ can bind internally in grooves or bind externally to phosphate groups of DNA to induce the formation of triple-stranded DNA and aggregation of nucleic acids.^70^ In addition to nonspecific binding, metal-mediated base pairing, in which two bases in complementary positions are bridged by a metal ion, also occurs. Metal-mediated base pairs in nature are well known, for example, T-Hg^II^-T and C-Ag^I^-C. In addition, many such pairs have been found by nuclear magnetic resonance and crystal structure analysis, and these structures usually have pyridine (Pyr) modifications or typical sequence characteristics. For example, the presence of Ag^I^ ions can promote the triplex d(T_10_PyrT_10_)⋅d(A_10_PyrA_10_)*d(T_10_PyrT_10_) to form a triple helix and increase its thermal stability, and metal-stabilized three-way junctions can be formed by incorporating Pyr into the center of a single-stranded nucleic acid.^71^

In addition, DNA nanostructures can respond to external environmental pH differences,^72,73^ which widely exist in biomedical engineering. For example, the tumor environment is acidic, while areas of bone regeneration are alkaline.^74,75^ Through ingenious design and careful implementation, DNA nanostructures can activate downstream reactions, thereby exerting their effects. A cytosine (C)-rich sequence usually maintains a linear structure in biological environments while folding into a stiff secondary structure, called the i-motif, in acidic environments.^76,77^ The i-motif is a tetrameric structure formed of two parallel duplexes through the intercalation of hemiprotonated CH^+^:C base pairs. In addition to slightly acidic conditions, Ag^I^ can provide protons to form i-motifs. Interestingly, guanine (G), when paired with cytosine, can form a special secondary structure in the corresponding G-rich sequence. Similar to an i-motif, a G-rich sequence can be held in the same plane to form a G-quadruplex (G4), which is induced by alkaline cations (such as K^+^ and Na^+^). Since the i-motif and G4 are complementary, they can be used for synergistic targeting, for example, the AS1411 aptamer with a G4 structure can be combined with acidic conditions.^78^

Taking advantage of the changes in nucleic acid structures in environments with specific ion conditions or pH values, researchers constructed various dynamic structures. Based on the pH sensitivity of the i-motif, Keum et al. reported a pH-sensitive drug delivery system to control the release of protein cargos.^79^ Kahn et al. provided a pH-responsive DNA hydrogel system, which could transition to a hydrogel at low pH and act as a drug carrier for controlled release.^38^ Ma et al. developed the pH-Apt-BiHCR technique for specific cell imaging and enhanced gene delivery by separation of the complementary chain followed by formation of the i-motif at low pH and stimulated the downstream cascade reaction.^80^

### Dynamic DNA structures based on strand displacement reactions

The abovementioned dynamic structures are based on the secondary structure of DNA. The unique sequence of ssDNA or stimulation via the external environment causes structural changes, resulting in various functions and applications. The basic structure of DNA is deoxyribonucleic acids connected by hydrogen bonds, yet competitive binding between the single strands of DNA can be generated by designing and adjusting the affinity of different single strands of DNA. Then, dynamic strand replacement can occur.^81^ The replaced single strand further induces the displacement of downstream DNA and performs the designed functions.^82^ This principle is applied in many artificial DNA reactions, including but not limited to HCR,^83,84^ catalytic DNA circuits (CDCs),^85,86^ and catalytic DNA hairpin (CDHs).^87,88^

HCR based on DNA hairpins is a typical representative artificial DNA strand displacement reaction. Specifically, hairpin HCR uses two hairpin DNAs (H1 and H2) and an initiator (I), and the sequence of each one should be complementary. For instance, the sequences classically designed by Robert are H1: abcb* and H2: c*ba*b*, I: a*b*. When the I is exposed to H1, the I has the opportunity to interact with H1 to form I·H1 and then release the b*c part of H1, which can bind competitively to H2 to form the I·H1·H2 complex and release the a*b* part of H2, resulting in the cascade reaction. Through this strategy, a 1 μmol·L^−1^ concentration of the hairpin system can efficiently detect a 0.1 μmol·L^−1^ concentration of the initiating strand, and by optimizing the structure design, the detection limit can decrease to ~5 nmol·L^−1^ initiating strand for a 50 nmol·L^−1^ hairpin system.^31^ Once HCR is triggered, the amplification reaction continues, and the speed of amplification is associated with the design of the hairpin structure. Amplification can be exponential when several activation sequences are designed, causing a cascade reaction based on DNA replacement. These HCRs can be used to construct biosensors for enhanced detection of nucleic acids, small molecules, or proteins, as well as for cell fishing.^89,90^ Guo et al. reported that a gold nanoparticle combined with a single-stranded DNA could be used for the sensitive determination of *Escherichia coli* O157:H7 through HCR and biotin-streptavidin signal amplification.^91^

Unlike that of HCR, the strand reaction of CDH and CDC can only be triggered by the initiating strand. When the initiating strand participates in the polymerization of the two hairpin structures, it is replaced and separated and then participates in the next round of reaction. This reuse is likely due to the consistency of the enzyme participating in the catalytic reaction, making the reaction process more controllable and milder. More specifically, the initiator can be a single DNA strand, a protein, or another biological molecule that participates in the reaction of the hairpin with the aptamer.^92^ The application of this kind of reaction can be significantly extended through such a combination.

### Dynamic DNA structures based on proteins or other substances

As genetic material, DNA can interact with other biological molecules, and this mechanism can be utilized in the design and application of various DNA structures. DNA polymerase participates in DNA replication, and the introduction of DNA polymerase into DNA structures can assist in other dynamic functions. Briefly, a circular DNA template is applied in a DNA polymerase-driven reaction, allowing prolonged linear strand extension. Hence, RCA is a simple yet highly efficient isothermal enzymatic amplification strategy to synthesize ultralong ssDNA. It benefits from the mild reaction conditions and its stability and efficiency in complex biological environments. Yang et al. combined rolling circle amplification (RCA) driven by DNA polymerase with aptamers to detect target proteins.^93^ Strategies utilizing RCA show promise for recruiting cells or detecting nucleic acid sequences, point mutations, and a number of viral genomes.^94,95^ For instance, Yao et al. combined the RCA strategy with a protein-targeted aptamer to accomplish cell envelopment and cell release, which was triggered by a DNA enzyme.^90^

Here, we briefly describe the chemical and structural basis of several types of dynamic DNA structures that are currently the main structures in use. It should be realized that the dynamic structure of DNA constitutes much more than these basic physical and chemical changes, and combined with its highly modifiable nature, dynamic DNA structures can perform even more functions.^96^ DNA can be connected with a variety of substances, such as biologically active substances (biotin, fluorescent groups, amino acids, and peptides) and inorganic substances (gold nanoparticles), through covalent or noncovalent bonds and finally play a role in downstream reactions.

## Applications of dynamic DNA nanostructures in the biomedical field

### Basic research on dynamic DNA nanostructures

**Fig. 5:**
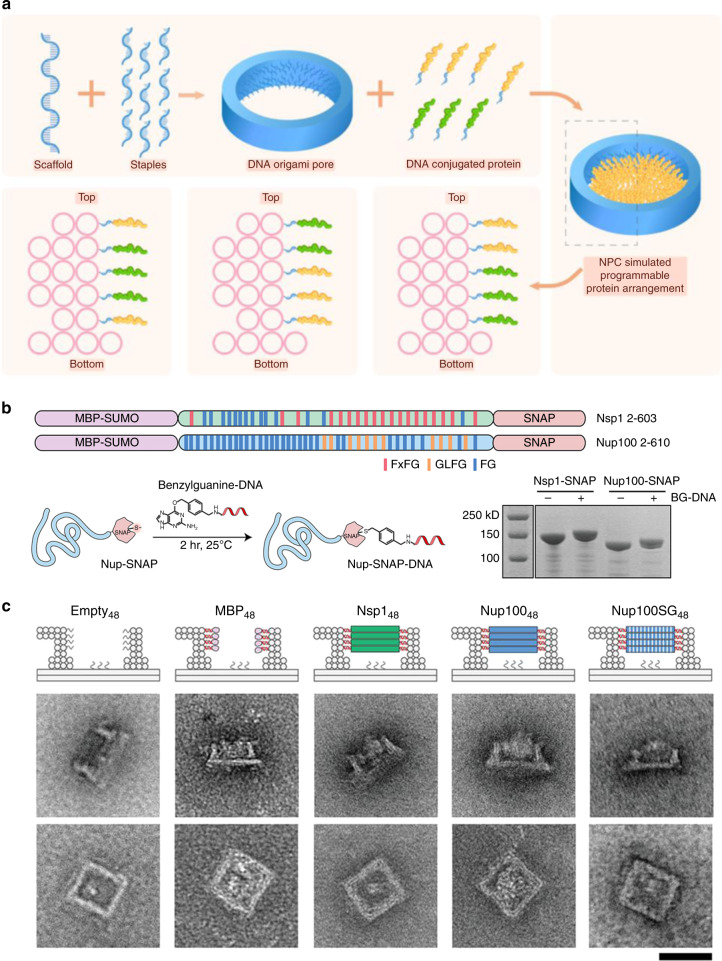
Assembly of nuclear pore complex mimics by DNA origami. **a** Schematic illustration of the fabrication of the DNA origami NPC mimic and its application in programmable protein arrangement. **b** As reported by Qi et al.^34^ two central channel nucleoporins of yeast origin, Nsp1 and Nup100, were expressed with a SNAP tag, which enables their conjugation with a benzylguanine-modified DNA oligo. **c** Illustration and TEM images of different protein-gated DNA origami NPC mimics. Scale bar = 50 nm. Copyright 2021, American Chemical Society

As described, the critical advantage of the DNA structure is its great editability and predictability of specific folding in experiments through in silico or theoretical design. However, breakthroughs in predicting protein structures, which are folded based on the constituent amino acids, have recently been made.^97^ In this application, DNA nanomaterials are used as tools to build structural foundations once proposed. This strategy is widely applied in various basic research fields, such as computer science (DNA computing, DNA circuit design, etc.),^98,99^ materials chemistry (DNA-based microscale arbitrary topological structures),^20,40–42^ structural chemistry (analysis of protein crystal structure assisted by DNA origami), and life science and biomedicine. In that process, dynamic DNA systems provide useful tools for biomimetic functions. Classically, DNA origami is employed as the structural basis for biomimetic structures and is used to investigate some structures that are difficult to reproduce by conventional methods, such as nuclear pore complexes (NPCs). NPCs are the most complicated protein complexes in eukaryotic cells and are composed of nucleoporins to form gateways that control molecular transport. The study of NPCs has far-reaching implications. However, native NPCs extracted from cells can be affected by the purification procedure, which results in differences from the native structure. Studies of individual nuclear pore proteins achieved by electrochemical methods are not reliable due to their separation from the physiological context. Hence, traditional approaches are limited. Lin et al. employed DNA origami to build a cylinder that mimics the dimensions, protein binding sites, density, and rules of native NPC channels and houses a specified number of nuclear pore proteins inside, thus constructing biomimetic nanopores in vitro (Fig. 5). Based on this platform, the structure and protein binding of nuclear pores can be precisely regulated. Hence, the characteristics and function of NPCs can be explored.^33,34^ Similarly, Dekker and Dietz designed a DNA origami ring based on a controllable structural foundation to decipher the behavior of intrinsically disordered proteins.^100^ In addition, a large number of researchers have employed DNA origami technology to explore the functions of proteins or complex dynamic processes relying on the precise controllability and modifiability of modifications. For example, this technology has been used to engineer lipid membranes with different sizes and shapes using DNA origami as the scaffold,^101^ explore lipid transfer between bilayers via DNA origami nanostructures,^102^ and program the dynamic assembly of viral proteins with viral genomic RNA via DNA origami nanostructures.^103^

In general, the main advantages of DNA nanostructures, including their strong structural editability, high yield, and modifiability after synthesis, make them an excellent platform for biomedical research. Thus, dynamic DNA nanostructures are widely utilized for some research objects whose structures, conditions, and contexts are difficult to control or reproduce. Considering the nature of DNA as a biomacromolecule, it has poor tolerance to complex chemical or environmental conditions compared with that of MOF structures. Hence, dynamic DNA nanostructures are promising in biomedical research.

### Drug transport and delivery with dynamic DNA nanostructures

**Fig. 6:**
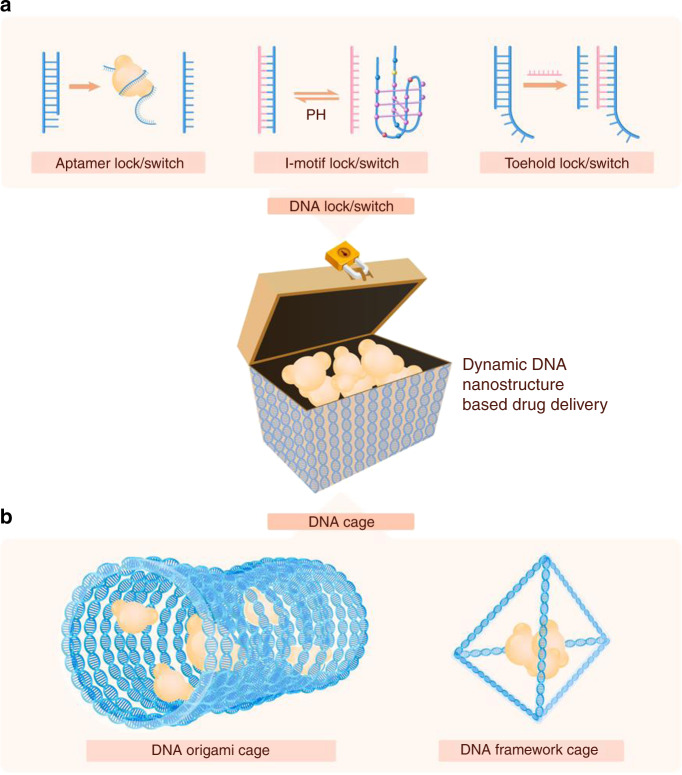
Drug transport and delivery with dynamic DNA nanostructures. The editability and physiochemical nature of DNA nanostructures allow various drug delivery strategies for dynamic and targeted delivery. **a** A repertoire of dynamic DNA nanostructures provides a stimulus-responsive lock and switch controlling cargo release. **b** DNA origami and DNA framework structures enable different cargo encapsulation strategies for cargo protection

Selectivity of drug delivery is achieved through two mechanisms: passive and active targeting. Passive targeting generally relies on the enhanced permeation retention (EPR) effect, which is dictated by the physical and chemical properties of the vehicle, such as its shape, size, and surface charge. For instance, different sizes of nanoparticles show different EPR effects and accumulate in different organs.^104^ In contrast, active targeting is achieved through various chemical or physical modifications to increase the selectivity of the carrier. Dynamic DNA nanostructures show natural advantages for active targeting design, mainly manifested in the following aspects (Fig. 6):

First, a wide variety of customized DNA nanostructures provide multiple options for drug delivery.^105–107^ A number of personalized microscale DNA structures have been efficiently synthesized. Correspondingly, a variety of nanoscale DNA structures have been widely applied and proven to realize controlled and efficient drug loading. One of the classic examples of drug transport by a dynamic DNA structure is the DNA cage encapsulating cytochrome *c* designed by Erben et al. Based on the chirality structure of the DNA double helix, cytochrome *c* is conjugated at consecutive positions along one edge of the DNA tetrahedron, and the position at which cytochrome *c* can be retained inside the cavity of the DNA tetrahedron is selected based on electrophoretic mobility by polyacrylamide gel electrophoresis (PAGE) and is then confirmed by nuclease digestion.^108^ Erben et al. provided a novel idea for encapsulating and protecting drugs through precise design and accurate control, and even loose framework DNA structures can provide great transport and protection. Recently, based on this observation, researchers have developed a large number of DNA nanosystems to protect cargo activity, control cargo release, and achieve active/specific targeting.^38,46^ Due to their higher nucleic acid density, DNA origami structures may theoretically provide better drug packaging. Moreover, DNA origami structures possess considerable structural redundancy. Hence, it is convenient to carry out beneficial chemical modifications, such as the addition of external stimulus-responsive modules. Ding’s group developed DNA origami self-assembled nanostructures to transport thrombin or antigen peptides, achieving targeted cancer therapy or cancer immunotherapy, respectively.^36,37^ Li et al. reported an RNase H-responsive framework nucleic acid for efficient microRNA unloading and intracellular deployment, and the nanostructure can significantly target histone deacetylase 5 (HDAC5) in bone marrow mesenchymal stem cells and promote osteogenesis.^109^ Furthermore, disordered structures such as DNA strands or particles constructed via RCA, a robust and easy method, are also applied in drug delivery.^110^ In RCA, DNA structures are generated by polymerization of DNA on a circular template through strand displacement, thereby producing a long polynucleotide strand with repetitive sequences. However, such disordered structures depend on passive rather than active targeting.

Second, in addition to options for drug delivery, various dynamic DNA nanostructures provide options for active targeting, which is achieved by aptamer sequences, ion-sensitive sequences, and chain replacement cascade reactions. Possible delivery systems have been proposed by combining therapeutic agents and specific context-responsive DNA switch structures. Examples include conjugation of a pH-sensitive i-motif sequence to gold nanostars to deliver DOX for combination cancer therapy,^111,112^ combination of a nucleolin-targeted aptamer sequence and efficient drugs such as melittin or thrombin for enhanced cancer therapy,^113^ loading of siRNAs with specific structures to efficiently transfer the siRNAs and regulate related biological behaviors,^114^ and enzyme cleavage to serve as a dynamic siRNA release mechanism for efficient gene regulation.^115^ Based on the response mechanism of dynamic DNA nanostructures to external stimuli, different drugs can be loaded to regulate physiological and pathological processes. Moreover, dynamic DNA switches can be loaded into other traditional materials, thus expanding the application of dynamic DNA nanostructures with active targeting ability.^38^

### Biosensors with dynamic DNA nanostructures

Biosensing applications are another main research field of stimulus-responsive DNA nanostructures. DNA sequences can respond to many external stimuli in addition to nucleotide sequences, ion concentrations, pH, and peptides. These response mechanisms provide the chemical basis for the construction of biosensors and allows the detection of the external context.^116–118^ Furthermore, DNA nanostructures possess special merits for sensing applications: (1) great controllability, as different structures have been developed to control the distance between sensors, thereby enhancing the specificity of detection;^119^ (2) intrinsic biocompatibility and great penetration;^120^ (3) the ability to be combined with hybridization chain reactions, by which dynamic DNA structures can accomplish signal amplification;^121^ (4) the ability to be conjugated to other materials, such as carbon nanotubes or polymers, to improve detection sensitivity;^122,123^ (5) the ability to be combined with various detection beacons by means such as fluorophore conjugation, metal ion conjugation, and chemical capture.^39,124^

**Fig. 7:**
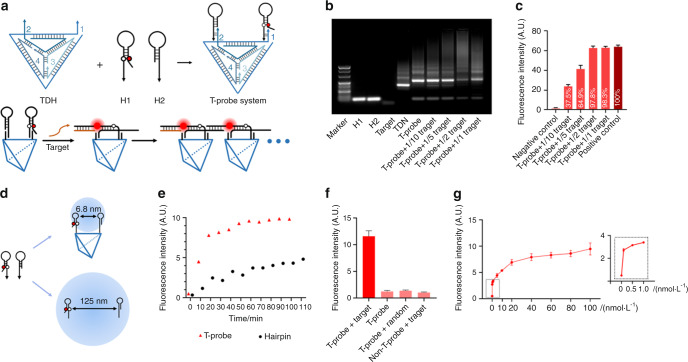
DNA hairpin-endowed biosensors. **a** Schematic illustration of a DNA tetrahedron (TDN)-based biosensor (T-probe) and its enhanced imaging of the target via HCR. The red dot indicates the Cy5 module; the white dot indicates the BHQ module. **b** Agarose gel electrophoresis image of the fabrication and HCR assay. **c** Fluorescence intensity responses of the T-probe exposed to the target at different molecular ratios. **d** Comparison of the reaction distance between naked DNA hairpins and the T-probe. **e** The time-dependent intensity of the T-probe suggests that it is a rapid sensor platform. **f** and **g** Specificity and sensitivity evaluation of the T-probe system.^125^ Copyright 2022, John Wiley and Sons

Importantly, the biosensing application of DNA nanostructures is mainly focused on detecting low concentration substances in a rapid manner. In a recent publication by Zhang et al., a DNA hairpin-loaded DNA tetrahedron was applied to achieve rapid detection of intracellular RNA and cell membrane proteins (Fig. 7).^125^ However, the downstream reaction after detection has attracted much more attention. Biosensors can be combined with other applications through different downstream reaction designs so that applications from detection reporting to cell capture can be realized.

### Tissue engineering with dynamic DNA nanostructures

The application of dynamic DNA nanostructures in tissue engineering is a new research field. With the development of gene therapy, researchers have devoted considerable attention to the prospects of functional nucleic acids, including but not limited to aptamers, mRNAs, miRNAs, and siRNAs, in the field of tissue regeneration. Nucleic acid-based devices offer a highly promising approach for the delivery of nucleic acids, due to their excellent biocompatibility, simple synthetic process, relatively high transfection efficiency, and low off-target effects.^126–129^ In our recent study, a model miRNA was delivered by an enzyme-responsive nucleic nanostructure for bone regeneration.^109^ miR-2861 shows high affinity for histone deacetylase 5 (HDAC5) and is beneficial for osteogenesis. Thus, it was loaded onto a tFNA via an RNase H-responsive sequence to construct a bioswitchable nanocomposite, benefiting from the outstanding internalization ability of the tFNA. When this nanostructure is delivered into bone marrow mesenchymal stem cells, the miRNA can be released from the nanocomposite in the presence of RNase H, which is abundant in the cytoplasm. The efficient unloading and intracellular deployment significantly enhance osteogenic differentiation of stem cells and bone regeneration in bone defect mice. Finke et al. developed an extracellular matrix mimic with a DNA hydrogel, which was designed by chemical crosslinking and enzymatic modification.^130^ The material is crosslinked by commonly available and low-cost salmon sperm DNA and modified by DNA polymerase I to endow cell compatibility and provide versatile attachment points. Cells can attach to the material through the cell-specific marker and release DNase I-mediated digestion products for further study. Moreover, the prepared DNA hydrogel was confirmed to promote the proliferation and differentiation of neuronal stem cells, resulting in the application of dynamic DNA nanostructures in nerve regeneration. The DNase I-responsive DNA hydrogel opens a new avenue for the development of dynamic DNA nanomaterials for use in tissue regeneration.

In addition, DNA assemblies can be synthesized as scaffolds for the precise positioning/organization of specific molecules. Kim et al. innovatively designed double-stranded DNA to organize a mineral carrier through the polymer-induced liquid precursor (PILP) method for enamel regeneration.^131^ It is worth noting that advances in dynamic DNA nanostructures in tissue engineering are only beginning, and multiple research groups are constantly contributing. More applications could be seen in the near future.

## Prospects and challenges in the use of dynamic DNA nanostructures in the biomedical field

### Rapid biological sample detection

The COVID-19 pandemic outbreak that was beginning by the end of 2019 was a tournament for more than the global public health system. The race between investments and endeavors from academia and industry and viral spread is attracting more attention now than any other time during this century. Among the related efforts, rapid identification and isolation of infected individuals are crucial. The mainstream technique, as well as the gold standard for COVID-19 detection, is polymerase chain reaction (PCR)-based detection of viral RNA. The PCR protocol itself is mature, widely applied, and trusted. However, for use in viral RNA extraction, RNA purification, reverse transcription, and cDNA amplification, PCR detection is limited due to its technical complexity, infrastructure dependence, prolonged detection time, and barriers to scale-up. While optimization of PCR protocols is allowing breakthroughs in rapid and facile virus identification, dynamic DNA nanostructures might also contribute. We take SARS-CoV-2 for example. The protein capsid and RNA genome allow multidimensional detection strategies for highly specific detection. A DNA aptamer has been reported to specifically bind to the receptor-binding domain of SARS-CoV-2’s spike protein with a binding affinity (K_d_) as high as 19.9 nM;^132^ DNA aptamers targeting nucleocapsids have also been reported,^133^ and multiple sites could be applied for the design of strand displacement systems to determine the RNA sequence of the viral genome.^134^ This viral detection strategy was highlighted by a recent publication by Wang et al.; an ORF1ab-targeting aptamer was applied to DNA tetrahedrons, which were loaded on a graphene membrane. Through an electromechanical approach, this design achieved direct ultrasensitive and rapid detection of SARS-CoV-2 RNA in less than four minutes.^135^ Considering that DNA nanostructures can be chemically and biologically synthesized on a large scale and the merits of simple fabrication and a mild detection environment, these dynamic DNA nanostructure-based rapid biomedical sensors could serve as potent tools early in future public health emergencies.^136^

In addition, DNA structures can be appended to other biomolecules to enhance functionality. Saka et al. covalently bound long DNA strands to antibodies, which bind to the target protein, and bound short fluorescence-labeled DNA strands to the long DNA strands, achieving a 5-180-fold intensification of fluorescence compared to that produced via the traditional protein antibody strategy.^137^ This fluorescence labeling strategy has been translated from academia to industry. Combined with DNA nanostructure processes enhancing tissue penetration, this labeling could be applied for diagnosis of oral mucosal diseases.

However, if promoted as a testing kit, several challenges remain. The most prominent challenge might be stability during distribution. Various laboratory applications of dynamic DNA nanostructures rely partially on the unstable nature of DNA molecules, which could, in contrast, cause problems for bedside applications. For instance, Pfizer’s well-acknowledged mRNA vaccine requires a cold chain for reliable distribution, which challenged its manufacturing and application in the early phase of the pandemic.

### Drug delivery

The delivery of drugs has remained a research hotpot for years, and researchers are envisioning drug loading vehicles with active targetability via delicate design. Regarding this application, DNA nanostructures have advantages and challenges. Some pioneers are pushing the limits of these applications. Ding’s group built a system applying DNA origami sheets as loading vehicles and aptamers that roll and lock the DNA sheet. When loaded with bioactive molecules, this vehicle could achieve different delivery goals by changing the targeting aptamer. In their first publication regarding vehicles equipped with the AS1411 aptamer, they performed tumor-targeted thrombin delivery, which suppressed tumor growth via in situ blood clot formation and circulation interruption. This system has undergone systematic biocompatibility and safety evaluation in minipigs and has been further extended as an antigen-presenting and siRNA delivery system for immunotherapy.^36,37,138,139^ These studies show the prospect of DNA nanostructures as delivery vehicles. However, the majority of research and applications are limited to in vitro experiments and in vivo exploration of simple delivery strategies. There are several factors that might impact these applications.

First, the chemical nature of DNA poses challenges for in vivo application of DNA nanostructures. The inherent scavenging of exogenous DNA damages the structure, and the existence of DNA enzymes in the circulation jeopardizes structural stability if serum stability were needed.

Secondly, the passive targetability of DNA nanostructures might limit their application to certain scenarios. For instance, tetrahedral DNA with an edge length of 21 bp accumulates rapidly in the liver and kidney once injected into the circulation via the tail vein, and the greatest amount is excreted in the urine.^24^ Renal clearance might be beneficial for some specialized applications, such as treatment of acute kidney failure.^140–142^ However, the metabolic characteristics would impact the serum concentration if other organs were targeted. In addition, accumulation in the liver and kidney could have potential toxicity.

Last, although the degradation product of DNA is regarded as biocompatible, the long-term biosafety remains to be evaluated. As DNA is the genetic material, there is a potential risk in the application of DNA materials, especially the current DNA origami folding structures with viral DNA scaffolds, inciting further debate regarding safety and esthetic issues.

However, new techniques are being developed to address the shortcomings mentioned above. DNA nanostructures prone to enzymatic digestion were scrutinized by researchers, and polymers were applied to meet the in vivo application requirement.^143–145^ Framework nucleic acid nanostructures are applied to avoid the potential hazard of introducing viral DNA into circulation.^113^ In brief, in situ application of nucleic acid vehicles for treatment in local and limited scenarios is currently regarded as more reliable than their application in more universal scenarios.

### Scaffold materials for tissue engineering

Tissue engineering has attracted tremendous and persistent attention. Several techniques have been translated from bench to bedside, such as the application of hydroxyapatite of various particle morphologies, structures, and doping in bone regeneration.^146^ DNA nanostructures can respond to external environmental conditions during tissue engineering. For example, pH-sensitive sequences can respond to the acidic pH in the fracture area, and aptamers can bind to the cytokines produced during tissue repair,^147^ thus providing sufficient imagination and design space for dynamic DNA nanostructures. The development of dynamic DNA nanostructures could bring novel insight to this research field. By combining the i-motif with a heat-sensitive polymer (poly(N-isopropylacrylamide), pNIPAM), researchers fabricated a hydrogel simultaneously sensitive to pH and temperature. The novel hydrogel was capable of reversible phase transformation from sol to hydrogel, therefore holding potential value for defect regeneration processes sensitive to pH and thermal changes, such as bone repairs.^38^ The combination of DNA systems has the potential to functionalize traditional scaffold materials.

On the other hand, dynamic DNA nanostructures are supporting the exploration of novel scaffold materials. Yao et al. combined a circular DNA template with an aptamer to realize the capture and release of BMSCs via the RCA technique.^90^ In addition, a recent study applied tetrahedral DNA to capture circulating tumor cells by aptamer targeting and magnetic bead enrichment.^148^ Equipping this design strategy into tissue engineering studies provides a new approach for the acquisition, homing, and enrichment of seed cells.

## Conclusion

Benefiting from multiple dynamic mechanisms, DNA nanostructures are revolutionizing traditional biomedical research and applications, with DNA-based biomedical sensors and drug delivery systems as pioneer products. Notably, the main merits of DNA nanostructures are regarded as the following: (1) biocompatibility; (2) unsurpassed editability and structural predictability at the nanometer scale; (3) versatility of chemical modifications; and (4) easy integration with other biomedical systems. These merits are the driving forces of various DNA nanostructure-based applications. For DNA nanostructures with base pairs serving as the elementary unit, the minimal editable scale is the length of a single base pair, approximately 0.34 nm, which is too large for atom-level and too small for macroscale regulation but is ideal for biomedical applications. These outstanding merits are leading breakthroughs in biomedicine. For basic research, dynamic DNA nanostructures provide a strategy for fabricating tools to investigate biostructures at the nanometer and submicrometer scales; for drug delivery, dynamic DNA nanostructures provide succinct and efficient stimulus-responsive approaches for active targeting, a further benefit to the excellent drug loading capability of DNA structures; for biosensor development, dynamic DNA nanostructures provide novel strategies for rapid and efficient biomolecular detection in vitro and in vivo; for tissue engineering, the repertoire of DNA nanostructures reacting with biomolecules offers a useful tool for gene and protein level regulation, while other possibilities are also under assessment, such as DNA hydrogels as tissue engineering scaffolds; for biomedical-related industrial considerations, dynamic DNA nanostructures provide a strategy capable of quick prototype production and easy scale-up, making it possible to handle health crises such as future epidemics and pandemics.

Compared to their traditional counterparts, such as polymers and metal particles, DNA nanostructures have unique merits. However, it is important to understand that DNA nanostructures and traditional materials could work in synergy to further extend their applications. For instance, dynamic DNA nanostructures can provide stimulus-responsive abilities, while polymers can serve as building blocks, providing a lower cost and a higher strength specific to different applications. This strategy can be applied for tissue regeneration processes requiring mechanical support, such as bone and joint repair. In addition, modifying DNA nanostructures with traditional materials endows DNA nanostructures with extended properties and applications: extending polymer brushes may boost the stability of DNA origami structures in an environment containing enzymes,^149^ and silica-deposited DNA hybrid structures can be ten times rougher than DNA alone.^150^

It is of similar if not greater importance to solve the unsolved challenges obstructing the application of dynamic DNA nanostructures from academia to industry, which include (1) their instability in the circulation and biosafety concerns; (2) the unclear proof of principle underpinning various biomedical effects of DNA nanostructures, causing concerns regarding long-term safety; and (3) the possibility that the instability and cost might jeopardize their applications in the real world. It is notable that DNA systems can be easily combined chemically with other traditional materials, such as polymers and proteins. These combinations could easily address some concerns, such as instability, cost, and uncertain biosafety. Researchers are also focusing on these concerns, for example, attempting to enhance DNA stability by chemical modifications or encapsulation by other biomolecules. Addressing and solving these challenges would strongly promote the further application of dynamic DNA nanostructures in the biomedical field.
